# Vets and Vaccines: A Discursive Analysis of Pet Vaccine Critique

**DOI:** 10.3389/fvets.2022.868933

**Published:** 2022-06-09

**Authors:** Pru Hobson-West

**Affiliations:** School of Sociology and Social Policy, School of Veterinary Medicine and Science, University of Nottingham, Nottingham, United Kingdom

**Keywords:** veterinary, ethics, vaccination, vaccine, immunization, sociology, public health

## Abstract

Critique of vaccination policy and practice has a long history, and social scientists and others have devoted significant efforts to understanding this phenomenon. This attention has only increased in light of the coronavirus pandemic, with public health concerns expressed about opposition to vaccination strategies. However, much less attention has thus far been devoted to understanding veterinary vaccine critique. This is problematic, given the central role of animals in the production and consumption of vaccines, and the existence of veterinary professional anxiety and international media coverage. The lack of existing literature may reflect a wider paucity of research on the veterinary profession; a paucity actively being challenged by new fields of veterinary anthropology and sociology. This short report is based on a discourse analysis of a UK campaign group, which questions aspects of companion animal vaccine policy. Findings suggest that the kinds of discourses used are similar to those made in the human vaccine domain: questions of risk, trust in expertise and imaginaries of science are thus not unique to human medicine. However, the article argues that some of the discourses identified are actually in line with wider social and cultural developments in healthcare. This argument has potential implications for veterinary professionals, as well as scholars interested in animal or human medicine. The article concludes by identifying future research trajectories, focused on further analysis of discursive practice, or the use of ethnographic observation to more fully understand the relationship between humans and non-humans, including animals and vaccine technologies.

## Introduction

This paper is part of a research topic on veterinary anthropology. This is defined as a new domain of research focused on understanding how veterinary knowledge and practices mediate changing relationships between humans and non-human animals. This commitment to focusing on veterinary knowledge and practice is also shared by sister fields such as veterinary sociology, where scholars are also belatedly recognizing the need for work which takes into account the role of animals and the veterinary profession in understandings of health, illness and medicine (1). As Brown and Nading (2) nicely summarize, “A view of health as more than human productively disturbs existing disciplinary settlements.” In both veterinary anthropology and veterinary sociology there is an urgent need to focus on the lived experiences of veterinary professionals and the animals they care for, for example *via* the use of classic ethnographic methods of observation. Some existing published accounts are in domains of high drama, for example the classic work by Morris (3) focusing on euthanasia; other contexts are apparently more mundane, for example work which focuses on the role of excrement in the way in which professional hierarchies are produced and maintained in farm animal practice (4). Existing work has also focused on the nuance of communication interactions between veterinarian and client, for example by utilizing video and observational methods to explore the iterative nature of veterinary consults (5), or, informed consent in companion anima neutering (6). Rather than focusing on the lived experience of veterinary professionals or client communication during consults, the contribution of this short report is instead to focus on discourse as the object of analysis. More specifically, it draws on gray literature to explore the discourses used by critics of veterinary vaccines. Whilst it is not the intention to draw a direct or formal comparison between the vaccination of children and companion animals (7), the main contribution is to identify key similarities in critical discourses in both human and animal medicine, and to identify avenues for further empirical research.

Sociologists have pointed out that vaccines should not just be regarded as just another medical technology; rather vaccination is positioned as one of the greatest success stories of modern medicine, science and modernity (8). Vaccination is thus highly symbolic, frequently constructed as the triumph of science over nature. As we have dramatically witnessed with the coronavirus pandemic, vaccine technologies are not just laden with historical significance, they also imbued with hope and expectation about society's ability to grapple with new diseases as they emerge, with Covid vaccines being frequently presented as our route back to “normality.”

Crucially, animals have a key role to play in the story of vaccine science and production. As stressed by those working within a One Health paradigm, animals (and how they and their habitat are treated by humans) play a key role in the emergence of diseases against which vaccines are developed. For example, scholars have stressed the way in which human-animal relations are implicated in the development of Covid 19 (9), and have previously shown how certain species become tarnished with “epidemic blame” (10). More specifically, vaccination as a technology has a long history, dating back at least as far as the experiments of Edward Jenner in England in 1796. Jenner showed that inoculating an 8 year old boy with cow pox legions helped to protect against smallpox: This helps explain the English term vaccine based on *vacca*, the Latin for cow (11). Non-humans are therefore entangled in this technology from the start. Animals also play a key role in scientific research and testing of new vaccines, standing in as models for human bodies. Indeed, reference to the development of life saving vaccines is often made when justifying the ethical use of animals as laboratory models (12). On the other hand, non-human animals are, according to Haraway, key “consumers of commodities” (13), and this includes vaccines.

The way vaccines are recommended and administered will depend on the role played by these animal consumers, for example as farm or companion animals. To promote sufficient depth, this short report will focus on companion animal vaccines. Some estimates have put the global pet medicine and vaccine market as worth over 40 billion dollars (14). For individual vet practices, vaccination can represent an important income stream, and an important opportunity for a wider health check with their animal patients. Animal owners are encouraged to adhere to a recommended schedule, although it should be noted that there has been active professional debate in recent years about the appropriate frequency of some vaccines, with a key industry body arguing against a “one size fits all” approach (15). More worrying for the veterinary profession, however, has been the development of concern expressed by clients. This has been summarized as a rise of so-called “anti-vax” sentiment, with headlines in major veterinary journals such as “*Don't let the anti-vaxxers win”* (16). The British Veterinary Association has also reported on findings from quantitative surveys claiming that 95% of UK vets have been questioned by their clients about the need for vaccination (17). Such professional anxiety is echoed in wider media coverage; For example, the UK saw media headlines in 2018 and 2019 including “*The anti-vaxxers have a new target in their sights – pets'* (18) or ‘*Sentencing their dog to death': how the anti-vax movement spread to pets”* (19), to “*Anti-vax spreads to animals as pet immunisations fall dangerously low”* (20).

This phenomenon is not confined to the UK. International media has also reported similar concerns about so-called “anti-vax” attitudes spreading by social media from human into veterinary medicine. In Canada, headlines warned “*Some pet owners believe vaccines give dogs autism. Vets say that's not true”* (21), and “*Anti-vaxxer ideology going to the dogs”* (22). In New Zealand, reports claim that “*Pet owners join anti vaccine movement”* (23), and that this is linked with other issues as in “*The rise of raw-food dog diets and pet anti-vaxxers”* (24). In Australia, headlines include “*Pet owners refusing to vaccinate animals over fears it will cause autism”* (25), and “*How one hippie town became the anti-vaxxer capital of Australia”* (26). Concern about the rise of veterinary vaccine critique has also been expressed by pet insurers (27).

Despite this professional and mass media interest, the topic of veterinary vaccine critique has not yet received social scientific conceptual or empirical analysis. Rather, a literature search only identified one short 2006 medical paper (7), arguing that vets should learn lessons from the MMR controversy. By contrast, and as demonstrated in the next section, social scientists have focused on the topic of human vaccination resistance, identifying useful theoretical concepts. The remainder of the article will identify some key similarities between human and pet vaccine critical discourses, before concluding with some suggestions for future ethnographic research.

### Human Vaccine Resistance

Despite the dominant success narrative associated with vaccination, there is a long history of organized opposition to vaccination policies and practices, dating back as far as the technology itself. Indeed, there were riots in English streets in the eighteenth century, with organized marches including the burning of effigies of Edward Jenner (28). The UK also plays a central role in more contemporary accounts of vaccine critique, with many blaming one individual—Dr Andrew Wakefield, and subsequent media coverage (7)—for encouraging this opposition, *via* his questioning of the safety of the MMR (measles mumps and rubella) vaccine at a press conference in London in 1998. Concerns about opposition to vaccination have also spread internationally: Indeed, Europe is the region with the highest level of vaccine hesitancy according to the Vaccine Confidence Project (29). This issue has also led to academic discussion about the extent to which the media can be blamed for vaccine hesitancy (30).

The Covid-19 pandemic has taken this media and academic interest in critique of vaccines to a new level of intensity. Some of this interest is in understanding individual decision making; others are trying to view critique of Covid vaccines as a social phenomenon. Whilst international publications express fear that Covid means routine vaccines are being missed (31), concerns were voiced early on in the crisis that existing vaccine skepticism could negatively impact on attitudes to Covid-19 vaccines (32). Indeed, media headlines continue to be dominated by discussion of the role of social media and celebrity figures who refuse vaccination. From a public health perspective, vaccine resistance is of interest due to the importance of herd immunity, and the assumption that that individual behavior has consequences for the community; for a sociologist interested in health and medicine, the claims and arguments underpinning all examples of vaccination resistance are worthy of critical analysis.

For example, drawing on research into organized vaccine resistance (as opposed to individual vaccine refusal), Hobson-West has previously argued (8, 33) against the dominant assumption that the debate is all about risk and risk perception. Rather, she argues, we need to focus more on questions of trust in medical professionals and those delivering public health messages. A decade on, other scholars lamented that the classic deficit model of public understanding of science (34) still unfortunately dominates in discussion of vaccine hesitancy, and still “shields science and government institutions from examining their own practices with respect to earning and maintaining public trust” (35). The consequences of this mismatch can be profound. As Kennedy has argued, there is a relationship between support for populist parties, as measured by the 2014 European Parliamentary elections, and vaccine hesitancy. Put simply, “Vaccine hesitancy and political populism are driven by similar dynamics: a profound distrust in elites and experts” (29). After analyzing online materials, Kata (36) labels this critique as postmodern, and uses this to explain why dominant (modernist) strategies of providing more information/health education are doomed to fail. More recently, Calnan and Douglass (37) have predicted that Covid-19 vaccine hesitancy is likely to reflect many of the same roots causes as concern with other vaccines.

As well as focusing on the central role of trust, detailed research has also helped complicate the assumption that critique is always “anti” science or anti public health. Rather, by analyzing the discourses of childhood vaccine campaign groups, Hobson-West (33) argued that, whilst resisting the “imperative of vaccination,” groups were actually conforming to other state and public healthcare messages, including the need to take personal responsibility for health. A similar claim has also been recently made in relation to HPV vaccines, such that, rather than expressing irresponsibility, mothers who questioned the vaccine did so by expressing “alternate responsibilities,” including a wider role in managing their teens' sexual health (38). More recently, Nurmi (39) has shown that rather than just rejecting vaccines, critics in Finland subscribe to a different image of microbes, which itself is more in line with a kind of multispecies approach to health. Such work confirms the value of detailed, qualitative research, to understand the nuance of, and interrelationship between, concepts like risk, trust, science, and nature.

### Method: Researching Pet Vaccine Resistance

Whilst not drawing on formal ethnographic observation, this article undoubtedly has autoethnographic origins. By training the author is a sociologist, but was based in a UK University Veterinary School for about a decade, where she still holds an honorary position. This experience includes teaching and research on the topic of animal health and welfare. As part of this professional role, informal fieldnotes were taken concerning the way vaccination is talked about by students, staff and the veterinary press. More specially, the motivation for this current paper was prompted by the following interaction:

“*Dear Dr Hobson-West. I hope you don't mind me contacting you out of the blue…The BVA [British Veterinary Association] has recently highlighted the concern in the veterinary profession about the rise of the anti-vax movement and anti-science rhetoric; it seems that more clients are questioning the need to vaccinate their pets…I wondered if you might be willing to speak at BVA Congress…and potentially offer some insights into how the veterinary profession can respond to those who are questioning the need for vaccination?” (personal fieldnotes, email communication)*.

This email was received on 24th May 2019. The sender works for the British Veterinary Association, the professional body for veterinary surgeons in the UK with over 18,000 members. Whilst this is just one email to one academic at one moment in time, it is reproduced here (with permission), to further illustrate the professional concern about resistance to pet vaccination. In response, in 2019 and 2020, the author carried out a documentary analysis of gray literature from the anglophone veterinary press and organized groups, in order to focus on the way in which claims were made and particular stakeholders constructed. This work is built on the assumption that the way arguments are made matters, if we are to fully understood social and political life, particularly in areas of controversy (40).

The analysis revealed a key role for one UK based campaign group, Canine Health Concern, established in 1994. The founder was Catherine O'Driscoll, a leading campaigner and author raising questions about companion animal vaccines. O'Driscoll has published several books and articles which were identified *via* internet searching. Printed and online material was coded by hand (without the use of a computer software package). A full discourse analysis is beyond the scope of this short report, but the next section briefly focuses on three key discourses or frames that show striking similarity with existing research on organized resistance to childhood vaccines. The implications of these parallels will be returned to at the end of this paper.

### Results: Shared Discourses of Resistance

“*So alongside reeling at the vaccine-related illnesses and deaths of countless dogs, I was also being exposed to similar from parents of human children….humans and dogs share blood, tissues, cells, immune systems and genes, and both species are capable of having inflammatory and autoimmune reactions to drugs and biologics” [(**41**), p. 107]*.

This quote is from “*The Tip of The Needle,”* a highly critical and reference heavy 485 page book, available to purchase online and authored by Catherine Driscoll of Canine Health Concern. As the above extract starts to illustrate, this group has themselves begun to draw parallels between human and animal vaccine critique. This section explores this comparison in more detail, using short extracts to illustrate each theme.

First, the online and printed campaign materials reframe the dominant way in which health risk is understood. In summary, risk is framed as either unknown, because of the claim that insufficient monitoring of disease and vaccine adverse reactions is done in the veterinary field; concealed, in that those in positions of authority such as veterinarians are not sharing what they do know; or is framed as non-random. From a sociological perspective, the latter is particularly intriguing. This is the argument that the risks animals face from disease, or from the recommend vaccines, are not equal between companion animals. Rather, risk is highly individualized. This is exemplified in the following quote from Canine Health Concern;

“*Your dog's diet will also determine whether the shots you give him are safe….Further, studies have shown that stressed people don't respond to vaccination – they fail to develop immunity… Genetic factors might also render vaccines harmful”* (42).

In this example, factors like diet, stress and genetics are argued to be influential in determining how an individual animal may react to a veterinary vaccine. This may sound simple, but is quite a fundamental critique, given the importance of recommended vaccine schedules for human and animal patients. This individualizing risk discourse is strikingly similar to the way in which human vaccine policy has been critiqued by organized groups, as exemplified in the following quote;

“*Creating and maintaining a reasonably sound, stable and healthy lifestyle is the best way to avoid illness and complications. Diseases do not strike randomly. [T]here would have to be underlying factors and weaknesses” [Informed Parent, cited in* *(**33**)]*.

Second, the materials on veterinary vaccination also suggest that trust in expertise is constructed as an important issue. However, crucially, what is negatively referred to as “blind trust” in the veterinary profession is constructed as highly problematic, as summarized in the following example;

“*We are conditioned to put others on a pedestal…because they have letters after their name….we need to take back our power and, with it, personal responsibility. We simply cannot afford blind trust anymore” [(**41**), p. 429]*.

The book*, the Tip of the Needle*, makes clear that the opposite of this negative blind faith, is a positive taking of personal responsibility for education and informed decision making. Here, then, rather than risk coming from the disease or the vaccine, trust itself is constructed as a source of risk. Once again, this is strikingly similar to discourses used in the childhood vaccination case. For example;

“*But remaining ignorant and trusting blindly can be the biggest risk of all. Only you really know what is the best decision for your child and hence the importance of learning enough to give you the ability to make that decision* [vaccination.co.uk website, cited in (33)].”

Thirdly, the discourse analysis also reveals similarities in the role and categorization of “science.” In the pet vaccine field, one interesting strategy was not to denigrate scientific or scientific research, as per the “anti-science” label used at the start of this article and in the email from the BVA. On the contrary, critical organizations try to do their own science, or what social scientists might label as “popular epidemiology” (43). For example, Canine Health Concern argues that;

“*Canine Health Concern Vaccine Survey was conducted in the 1990s and involved 3,800 dogs. Our findings were astonishing, and confirmed that there is a high likelihood of your dog becoming ill within three months of a vaccine event”* (44).

In this example, the group was motivated to collect their own data, and used this to guide their campaigning. This is very similar to arguments made by parent groups set up to campaign on issues of childhood vaccines including MMR, and exemplified in the following extract.

“*we set up a computer database and started to put the parents' details in…It's only through building up that database that we started to identify bowel disorders, epilepsy, autism, speech and learning difficulties, and all these things that parents were reporting”* [JABS cited in (45)].

Overall, this section has drawn direct parallels between the way in which concepts like risk, trust and science are constructed in organized vaccine resistance. In short, key similarities are evident between the human and animal healthcare fields. What this means for social science and humanities scholars, and for the veterinary profession, will now be considered.

## Discussion

This paper began by recognizing the value of the emerging fields of veterinary anthropology and veterinary sociology, and highlighting the existing lack of attention to the topic of vaccination. This is despite the symbolic weightiness of vaccines, and the central and varied role that non-human animals play in both the production and consumption of vaccine technologies. By contrast in the UK and elsewhere, professional and mainstream media attention has been devoted to expressing concern over an apparent rise in client questioning of veterinary vaccines. The aim of this short article was to provide a preliminary analysis of vaccine critical materials in the companion animal domain. Using extracts from printed and online material, it was shown that vaccine critique takes a similar discursive form to organized critique of childhood vaccination. For scholars keen to break down barriers between the study of human and animal medicine, this finding should not be seen as surprising. Afterall, “vaccines are vaccines,” whatever species their recipient: The fundamental questions of risk, trust in expertise and imaginaries of science are clearly not unique to human medicine.

To explore this finding a little further, previous research in the human field has argued that whilst vaccine critique may appear dangerous or “other,” it could be regarded as in line with other trends which health professionals and policy makers themselves have a role in encouraging. For example, the idea of risk as non-random or individualized is arguably in line with wider trends in personalized medicine/care and geneticization. In terms of trust, critics who argue in favor of personal responsibility and not “trusting blindly” are somewhat echoing the expert patient agenda. Likewise, engaging in a form of scientific data gathering or popular epidemiology could be said to conform to wider trends in citizen science (45). A similar claim can now be posited for veterinary vaccine critique: That some of the discourses are in line with existing trends within contemporary veterinary medicine and science. If correct, then this analysis has significant implications for the veterinary community. In short, it is likely to be counterproductive to dismiss campaign group such as Canine Health Concern or their members as “anti-vax,” or “anti-science,” or to see vaccine critique simply as a challenge of communication (7). Furthermore, it is not sufficient to automatically blame social media for the spread of such critical ideas. For those who regard vaccination as the obvious choice for animal health and welfare and in line with scientific orthodoxy, expressing concern about media debate is understandable; however, sociological research on human vaccines tells us that individuals are able to exhibit multiple “layers of reflexivity” regarding vaccination and social media (46).

Given the relative lack of existing research, further empirical study using techniques of ethnographic observation would now be valuable. This could compare how vaccination works in the human and animal clinic, and the extent to which practices differ in the farm animal domain. In terms of theoretical frameworks, such work could maintain a “human centered approach” (47), and study, for example at how veterinary knowledge is transformed into normative practice, how vets articulate a counter-narrative, or the extent to which vets themselves may experience anxiety around risk (48). Or, it could adopt a more than human ethnography or multispecies perspective, taking into account the role of animals or microbes as actants, or the way all species are engaged in “embodied life and movement” (49). Alternatively, like the present paper, future empirical work could study discourse but focus on the discourse of animal owners, rather than organized resisters, and involve more detailed exploration of what Nurmi (after Enticott) identifies as “lay immunologies” (39). In particular, it would be interesting to use in-depth interviews to explore the relative impact of the vaccine critical discourses identified in this short report, on lay immunologies.

Going forward, scholars from different sub-fields will no doubt identify their own research priorities. However, I would advocate specific attention as to whether and how experiences in animal healthcare can “loop back” to impact on human healthcare practices, and vice-versa. Or, to take this point further, how human and animal health and healthcare are co-constructed or interdependent (50). Indeed, social scientific empirical work on other topics has shown how animal owners blur boundaries between human and animal health imaginaries, for example by adopting shared disease categories between themselves and their animals, even sharing technologies or adopting shared practices [e.g., (47, 51)]. Interviews or observation with animal owners could therefore explore whether this holds true for images of preventive healthcare such as vaccination. To conclude with a provocation, is it possible that existing choice in the *veterinary vaccine* field, calls to avoid a “one size fits all” approach and the possibility of titer testing to ascertain individual immunity, could impact on expectations or demand for more choice or consumerism in the *human vaccination* arena? This is just one example of how the exciting research trajectories opened up by veterinary anthropology and veterinary sociology should benefit wider work in the social science of health and medical humanities.

## Data Availability Statement

Publicly available datasets were analyzed in this study. The data that support the findings of this study are online media articles, published books, and published journal articles. These data were derived from sources that are provided as references at the end of this article.

## Author Contributions

The author confirms being the sole contributor of this work and has approved it for publication.

## Funding

This work was supported by the Wellcome Trust *via* a grant to the University of Nottingham [204843/Z/16/Z] For the purpose of open access, the author has applied a CC BY public copyright license to any author accepted manuscript version arising from this submission. The author was supported by a Wellcome Trust Prime Scholarship.

## Conflict of Interest

The author declares that the research was conducted in the absence of any commercial or financial relationships that could be construed as a potential conflict of interest.

## Publisher's Note

All claims expressed in this article are solely those of the authors and do not necessarily represent those of their affiliated organizations, or those of the publisher, the editors and the reviewers. Any product that may be evaluated in this article, or claim that may be made by its manufacturer, is not guaranteed or endorsed by the publisher.
